# Augmented reality for guiding biopsies in rare retroperitoneal fibrosis cases

**DOI:** 10.1097/MS9.0000000000003914

**Published:** 2025-09-17

**Authors:** Soumiya Nadar, Muneeb Saifullah, Maliha Khalid, Muhammad Talha, Aminath Waafira

**Affiliations:** a Department of Medicine, Tbilisi State Medical University, Tbilisi, Georgia; b Department of Medicine, King Edward Medical University, Lahore, Pakistan; c Department of Medicine, Jinnah Sindh Medical University, Karachi, Pakistan; d School of Medicine, The Maldives National University, Malé, Maldives

**Keywords:** augmented reality, image-guided biopsy, precision medicine, retroperitoneal fibrosis

## Abstract

Retroperitoneal fibrosis (RPF) is a rare immune-mediated fibro-inflammatory disorder that often encases retroperitoneal structures, posing diagnostic and procedural challenges. Conventional image-guided biopsy techniques using CT or MRI may lead to missed lesions, sampling errors, and repeated procedures. Augmented reality (AR) platforms, such as Microsoft HoloLens-based systems, overlay 3D patient-specific anatomy in real time, enabling clinicians to visualize biopsy needle trajectories without dividing attention between monitors and the procedural field. Phantom studies have shown AR guidance can reduce soft tissue biopsy error from 1.52 to 0.75 cm, and lung lesion biopsy error from 1.12 to 0.62 cm. Despite promising accuracy gains, challenges remain in atypical RPF cases, including irregular lesion morphology, proximity to vital structures, and diagnostic ambiguity. Prospective multicenter studies are needed to confirm AR’s clinical utility, assess cost-effectiveness, optimize training, and ensure practical and ethical integration into routine biopsy practice.

*To the Editor,*

Retroperitoneal fibrosis (RPF) is a rare immune-mediated condition characterized by chronic inflammation and fibrosis in the retroperitoneum. The ureters, aorta, and vena cava are retroperitoneal structures that are frequently encased and compressed as a result of this fibrotic process. Although the actual prevalence of RPF is unclear, it is typically calculated to be 1.4 cases per 100 000 people annually. Over 90% of patients present with rather vague discomfort, which is the most common presenting symptom. This discomfort is usually described as a dull ache in the abdomen, back, inguinal, or flank areas^[1]^.

Traditional image-guided biopsies using CT and MRI may lead to missed lesions or sampling errors. These require multiple imaging sessions, increasing radiation exposure and procedure time. Studies presenting augmented reality (AR)-guided biopsy systems use Microsoft HoloLens to overlay 3D models of the patient’s anatomy in real time^[2]^. This allows the clinician to see exactly where the biopsy needle is going inside the body, without relying solely on mental mapping. As a result, they avoid splitting their attention between the procedure site and external monitors by looking at integrated images during the procedure.

Phantom studies simulating soft tissue and lung lesions demonstrated that AR guidance significantly improved biopsy accuracy. The average biopsy error for soft tissue lesions was reduced from 1.52 (CT-only) to 0.75 cm (AR-guided). Lung lesion biopsy error dropped from 1.12 (C-only) to 0.62 cm (AR-guided)^[2]^. Another study, consisting of an AR system of HoloLens 2, the Unity game design software, and an optical motion capture camera system, studied the accuracy of prostate biopsies. With a mean targeting error of 2.94 ± 1.04 mm after calibrating, it was clinically acceptable for prostate biopsy^[3]^.

Despite AR’s capabilities, there are several concerns that need to be addressed. A 2006 case report described a unilateral perirenal RPF without ureteral encasement, closely mimicking malignant and other infiltrative lesions on imaging. The atypical location, irregular lesion margins, and proximity to major vessels reduced diagnostic confidence. Despite CT and MRI, repeated percutaneous biopsies were required before confirming its benign fibrotic nature. This case illustrates anatomical and procedural challenges that persist even with advanced visualization^[4]^.

To fully realize the benefits of AR and mitigate its limitations, multicenter prospective studies are essential. These studies need to evaluate not only improvements in ultrasound skills, procedural efficiency, and safety measures but also address practical issues such as implementation costs, skill retention, cost-effectiveness, and ethical considerations^[5]^. By addressing these key considerations, we can not only optimize the benefits of AR but also develop user-friendly and cost-effective AR solutions that can be widely adopted. Our work is in line with the TITAN Guidelines on the need for transparency in AI use in healthcare^[6]^.

## Data Availability

No data were generated for this manuscript.

## Research registration unique identifying number (UIN)

1. Name of the registry: Not applicable. 2. Unique Identifying number or registration ID: Not applicable. 3. Hyperlink to your specific registration (must be publicly accessible and will be checked): Not applicable.
